# Characterization of Combinatorial Polymer Blend Composition Gradients by FTIR Microspectroscopy

**DOI:** 10.6028/jres.109.014

**Published:** 2004-04-01

**Authors:** Naomi Eidelman, Carl G. Simon

**Affiliations:** American Dental Association Foundation, Paffenbarger Research Center, National Institute of Standards and Technology, Gaithersburg, MD 20899-8546; National Institute of Standards and Technology, Gaithersburg, MD 20899, USA

**Keywords:** biomaterials, combinatorial methods, combinatorial materials science, FTIR microspectroscopy, high-throughput assay, low-e glass, poly(lactic acid), polymer blends

## Abstract

A new FTIR technique was developed for characterizing thin polymer films used in combinatorial materials science. Fourier transform infrared microspectroscopy mapping technique was used to determine the composition of polymer blend gradients. Composition gradients were made from poly(L-lactic acid) (PLLA) and poly(D,L-lactic acid) (PDLLA) in the form of thin films (6 cm × 2 cm) deposited on IR reflective substrates. Three composition gradient films were prepared and characterized. The results demonstrate the reproducibility and feasibility of a new, high-throughput approach for preparing and characterizing polymer composition gradients. The combination of composition gradient film technology and automated nondestructive FTIR microspectroscopy makes it possible to rapidly and quantitatively characterize polymer composition gradients for use in combinatorial materials science.

## 1. Introduction

Polymer blending is designed to generate materials with optimized chemical, structural, mechanical, morphological and biological properties [1–4]. Preparation of individual ratios of blended polymers requires many combinations and each has to be individually characterized. However, polymer blends readily lend themselves to study via combinatorial methods [5,4]. Composition gradients of polymers can be created that allow a wide range of polymer blend compositions to be studied on a single specimen. However, current methods for characterizing polymer composition gradients with traditional FTIR spectroscopy [5] are slow and inefficient. Therefore, we combined the gradient polymer blends combinatorial approach with Fourier Transform Infrared (FTIR) microspectroscopy mapping as the characterization tool to achieve a high-throughput technique for characterizing numerous variations of polymer blends rapidly and efficiently.

PLLA and PDLLA are biodegradable polymers used in tissue engineering for medical applications. They are chemically identical but differ in their crystallinity (PLLA is crystalline, PDLLA is amorphous) [6]. Blends of these polymers vary in their crystallinity and morphology. Thin film composition gradients of poly(L-lactic acid) (PLLA) and poly(D,L-lactic acid) (PDLLA) blends were prepared on low emission reflective slides and were characterized with reflectance FTIR microspectroscopy. Subjecting the thin film coated reflective substrates to FTIR microspectroscopy in reflectance mode, resulted in reflection-transmission spectra (FTIR-RTM). A calibration curve was constructed using discrete blends of PDLLA and PLLA, and was applied to the composition gradients using a sophisticated suite of software packages to yield a reliable, efficient, high-throughput method for characterizing polymer composition gradients. The non-destructive nature of the FTIR-RTM allows characterized specimens to be used for additional experimentation. Herein, we present this new method for characterizing the composition of polymer composition gradients using FTIR-RTM.

## 2. Materials and Methods

### 2.1 Sample Preparation

Pure polymer solutions of PLLA (*M*_w_ = 300 000; Polysciences, Warrington, PA) and PDLLA (*M*_w_ = 330 000 to 600 000; Polysciences, Warrington, PA) were prepared by dissolving the respective polymers in chloroform (1 % by mass fraction). Discrete composition films [(0, 25, 50, 75, 100) % by mass fraction] were prepared by mixing PLLA and PDLLA solutions in the appropriate ratios and then spreading the mixtures on low-emissivity (low-e) reflective glass slides (2.5 cm × 7.5 cm, Kevley Technologies, Chesterland, OH) using a home-built flowcoater [7]. Low-e highly reflective microscope slides were used because their mirror-like coating reflects the infrared beam back through the polymer film to yield reflection-transmission spectra, which are equivalent to “normal” absorption spectra after converting them to absorbance (without any mathematical correction) [8–9]. In addition, their large dimensions (7.5 cm × 2.5 cm), the microtexturing of their surface that improves wetting [9] and their resistance to organic solvents and water make low-e slides perfect substrates for macro size polymer films that can be characterized with FTIR-RTM. The dimensions of the discrete composition films were approximately 2.5 cm × 2.5 cm. The thicknesses of the centers of 100 % PLLA film (384 ± 34) nm and 100 % PDLLA film (162 ± 12) nm were determined by atomic force microscopy (in tapping mode, Dimension 3100 Nanoscope IIIa, Veeco Instruments, Inc., Woodbury, NY). Three PLLA-PDLLA composition gradient films (6 cm × 2 cm) were prepared on low-e glass slides using a three-syringe pump system as described in Ref. [7,4] and were characterized in order to establish reproducibility. The gradient films and the discrete blend films were melted at 200 °C (above the *T*_m_ of PLLA) for 5 min and then annealed at 120 °C (above *T*_g_ of both polymers) for 8 h under nitrogen to allow spherulites to form in the PLLA and to remove residual solvent.

### 2.2 FTIR Microspectroscopy: Construction of the Calibration Curve

The FTIR-RTM measurements were performed with a Nicolet Magna-IR 550 FTIR spectrophotometer interfaced with a Nic-Plan IR microscope. The microscope is equipped with a video camera, a liquid nitrogen cooled-mercury cadmium telluride (MCT) detector (Nicolet Instrumentations Inc. Madison, WI, USA) and a computer-controlled mapping translation stage (Spectra-Tech, Inc., Shelton, CT, USA) which is programmable in the *x* and *y* directions. The spectral point-by-point mapping of the films was done in a grid pattern with the computer-controlled microscope stage and the Atlus software package (“acquisition package”). Spectra were collected from 4000 cm^−1^ to 650 cm^−1^ at a spectral resolution of 8 cm^−1^ with 32 scans and a beam spot size of 400 μm × 400 μm. The spectra were ratioed against the background of uncovered regions in the low-e glass. Background was obtained during the mapping usually after every 10 spectra to compensate for slight changes in the chamber atmosphere.

**Fig. 1:**
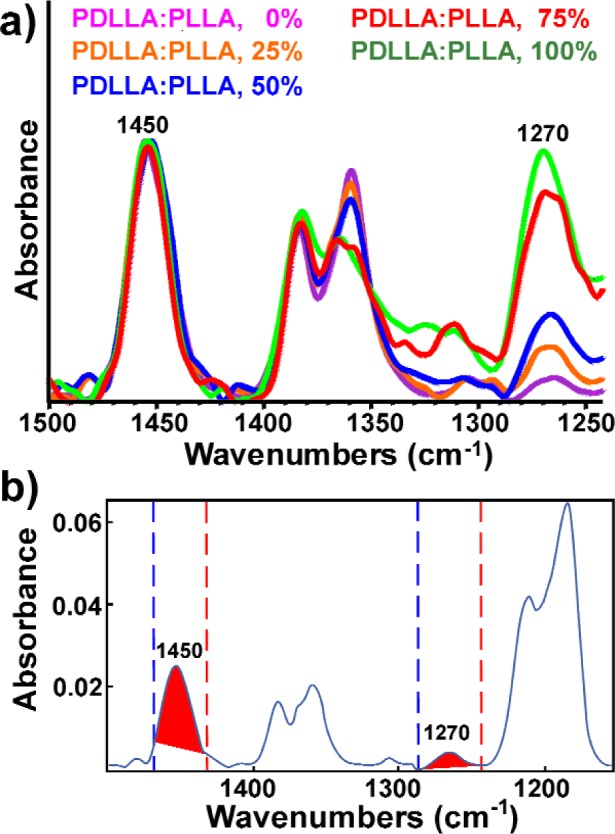
a) Spectra obtained from discrete composition films normalized to the 1450 cm^−1^ peak. b) 1450 cm^−1^ and 1270 cm^−1^ peaks in the respective spectral regions used for peak ratios.

FTIR maps were obtained from three 100 % PLLA and three 100 % PDLLA films, one 25 % PDLLA film, one 50 % PDLLA film and one 75 % PDLLA film (mass fraction). The map spectra were baseline corrected between 1500 cm^−1^ and 1155 cm^−1^ and total absorbance maps in this spectral region were obtained. In order to normalize for differences in the thickness of the films, the absorbance maps were processed as ratios of the 1270 cm^−1^ peak area (between 1287 cm^−1^ and 1244 cm^−1^) to the 1450 cm^−1^ peak area (between 1470 cm^−1^ and 1432 cm^−1^). The 1270 cm^−1^ peak was dependent on PDLLA concentration and became larger as PDLLA concentration increased. The 1450 cm^−1^ peak was not dependent on blend composition and was used as an internal standard (Fig. 1). The maps were displayed as color contour maps.

**Fig. 2:**
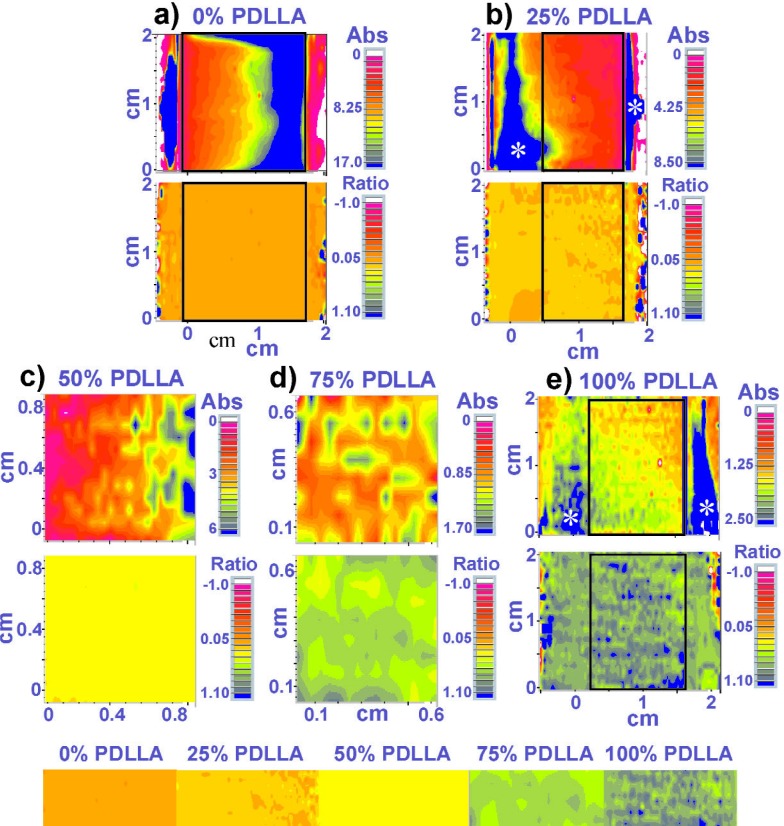
Absorbance (top) and ratio (bottom) maps of five discrete composition films. For the absorbance maps, blue indicates the highest absorbance and pink represents the lowest. The absorbance of the 0 % PDLLA film is higher than that of 100 % PDLLA film, which indicates that the 0 % PDLLA film is thicker than the 100 % PDLLA film. Note that the color range of the absorbance maps of discrete blends containing higher % PLLA is larger, which is an indication of thicker films. For the ratio maps, orange indicates the highest % PLLA and blue indicates the highest % PDLLA. White stars on the blue regions in the absorbance maps indicate thicker regions. Note that the absorbance and ratio maps in panel 2e appear also in Fig 3a. The color bar in the bottom was assembled from the middle parts of the ratio maps of the discrete composition films.

**Fig. 3:**
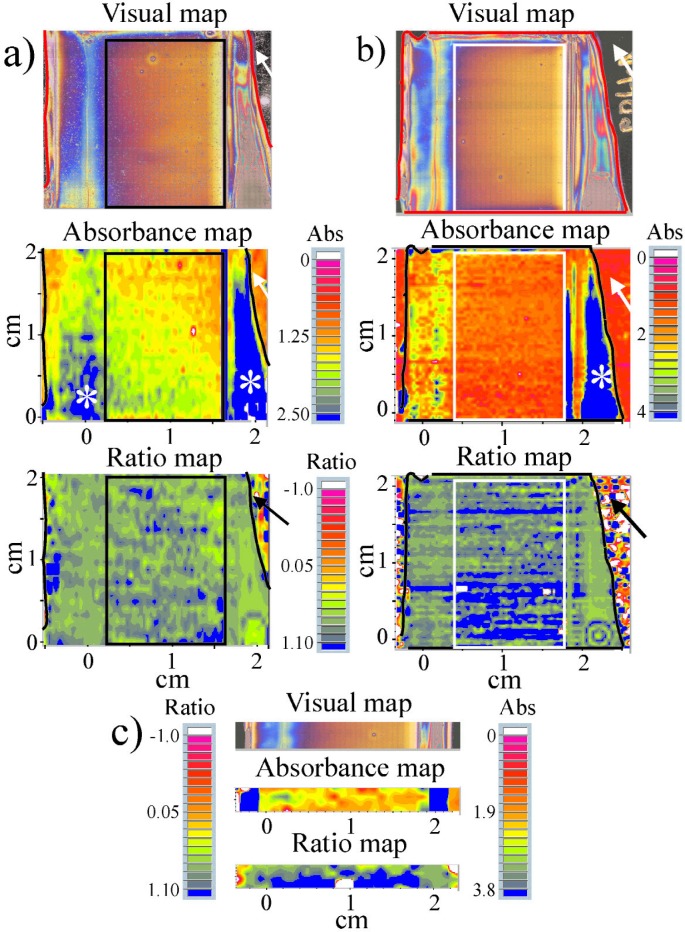
Visual, absorbance, and ratio maps of three 100 % PDLLA films. Only the middle of film #3 was mapped (panel c). The arrows (panels a and b) point to bare regions of the slide that are not covered with polymer film. The colors in these bare areas seen in the ratio maps are artifacts of ratioing spectra of the reflective glass substrate. White stars on the blue regions in the absorbance maps indicate thicker regions.

The film preparation method produced 100 % PDLLA films that were thinner than the 100 % PLLA films; hence, PLLA-rich discrete films and PLLA-rich regions in the composition gradient films were usually thicker than the PDLLA-rich films. This can be seen qualitatively in the absorbance maps of the discrete blends (Figs. 2 and 3) and quantitatively in the area of the 1450 cm^−1^ peak in the discrete blends (Table 1).

Initially, it was not possible to translate the pixel values from the color maps into actual values of percent PDLLA because the numerical data from the maps could not be exported from the acquisition software. The acquisition software is able to process and ratio spectra to create the color maps, but it is not possible to access the actual numerical values used in the final maps. Thus, we chose representative spectra from each map and processed them manually using traditional approaches via the Omnic-Altus package (Method A). However, we developed a new, high-throughput method (Method B) to translate the pixel values from the color maps into values of percent PDLLA. By adjusting the ISys software package (Spectral Dimensions, Inc., Olney, MD) to import the acquisition software maps, the imported maps can be processed in the same spectral range and peak ratios as in Method A, and the numerical values for each pixel in the map are accessible. Access to the numerical values allows rapid and accurate construction of calibration curves as well as a thorough characterization of the composition gradients. We will first discuss the traditional method and then present the newer, high-throughput method. It is useful to consider the traditional method (Method A) first since the accuracy of the newer method (Method B) is established by comparison to the results of the older method.

Originally, 4 to 14 representative spectra (selected by visual estimate of the distribution of the colors in the maps) were extracted from each of the discrete composition peak-ratio maps for use in the calibration curve. The number of spectra extracted depended on the homogeneity of the ratios in the maps. As can be seen in Fig. 2, the colors in the ratio maps of the films with 0 % (Fig. 2a), 25 % (Fig. 2b) and 50 % PDLLA (Fig. 2c), were quite homogeneous, therefore the number of spectra that were used for calibration were: 5 from the 0 % PDLLA (5 light orange out of 1612 spectra), 5 from the 25 % PDLLA (2 light orange and 3 orange-yellow out of 1534 spectra) and 4 from the 50 % PDLLA (4 yellow out of 288 spectra). Five spectra were extracted from the 75 % PDLLA control (Fig. 2d, 1 yellow-green, 1 light green, 1 medium green and 2 dark green out of 120 spectra) and 14 spectra (1 medium green, 2 dark green, 3 green-gray, 4 gray-blue and 4 blue, out of 1716 spectra) were extracted from the 100 % PDLLA film (Fig. 2e). Because the 100 % PDLLA film shown in Fig. 2e was quite thin, the absorbance of the 1450 cm^−1^ peak was low, especially in the middle (peak area average of 0.06 versus almost double the area of the 75 % film and ten times fold of the average area of the 50 % PDLLA, see also Table 1), and there were regions where the signal to noise ratio (SNR) was low. In these regions, the contribution of the background water vapor was significant in the edges of the 1450 absorption peak, resulting in larger errors in the peaks ratios. Therefore, eight spectra from the ratio maps of two additional 100 % PDLLA films (prepared on the same day, Fig 3b, c) were extracted and the peaks ratios were measured and included in the calibration curve. Note the similarity in the visual images of all three films.

For each of the extracted spectra, the areas (without additional baseline correction, Fig. 1) of the 1270 cm^−1^ peak (between 1287 cm^−1^ to 1244 cm^−1^) and the 1450 cm^−1^ peak (between 1470 cm^−1^ to 1432 cm^−1^) were measured manually with the peak area tool available in the Omnic software. These peak ratios were used for the calibration curve. The narrow spectral region used for the 1450 cm^−1^ peak (1470 cm^−1^ to 1432 cm^−1^) was chosen to avoid noise from the atmospheric water, which was problematic when the films were thin. The colors of the pixels in the maps from where each extracted spectrum was taken were recorded so colors from the maps could be assigned composition values (see color code in Fig. 2).

Next, the high-throughput method (Method B) for translating the pixel values from the maps into percent PDLLA using ISys (processing software) will be presented. As mentioned above, the numerical data from the color maps cannot be exported directly from the acquisition package. However, the processing software is able to import the spectral maps generated by the acquisition package. Based on our requests, the developer of the processing software made proprietary modifications so that it was possible to import data from the latest version of the acquisition package that was used to obtain the maps in this study. This allowed us to correctly process the exported values from the processing software and to match the manual values obtained from Method A to those acquired by Method B. The absorbance maps of the discrete composition films were imported into the processing software and were processed as peak ratio maps in the same spectral regions as was done earlier with Method A. The processing software has a useful feature that allows zooming in any area of interest in the map while truncating the rest of the map, thus providing the option to get immediate averages and standard deviations from any region of the map. This feature was used to remove edges containing bare spots and thick regions from maps that were collected from whole films of discrete composition blends. The 75 % and 50 % PDLLA maps (Fig. 2c, d) did not need to be cropped, because spectra were collected only from the middle of the films. The rectangles on the maps in Figs. 2 and 3 mark the truncated regions on which the integration was done in the processing software. The number of spectra that were included in each integration, appear in Table 1. The average peak ratios obtained from these regions were used for the calibration curve. Just thin edges of the 0 % PDLLA map that contained bare edges of the reflective slide were cropped (rectangle on the map in Fig. 2a) while larger regions were cropped from the 25 % and 100 % PDLLA maps (Figs. 2b, 2e and Figs. 3a, 3b). These regions were chosen so as to avoid including significantly thicker regions in the outer sides of the films whose absorbance was ≈20 times higher than in the middle region (see blue regions with white stars in the absorbance maps in Figs. 2b, 2e and Figs. 3a, 3b). These steps were taken, because (a) no such regions existed in the gradient films (see visual maps of the gradients, Figs. 5–7); (b) the SNR was significantly lower in the middle thin regions of the 100 % PDLLA films than in the thick regions, and (c) the calibration curve was constructed from controls that had to be as similar as possible to the various regions in the gradient films.

The averages of the 1450 cm^−1^ peak areas of the discrete composition films that were calculated by the processing software (Table 1) and that were used later for the calibration curve indicate large differences in the thickness of the films. Only negative values of peak ratios (obtained most probably from bad spots) were excluded from the averages. Four ratios out of 780 were excluded from the 25 % PDLLA film, and 77 out of 1802 were excluded from the 100 % PDLLA #1 (Fig. 3b). No ratios were excluded from the other discrete maps. While four spectra from the strip map of the 100 % PDLLA #3 (Fig. 3c) were included in the manual calibration curve, only the two maps seen in Fig. 3a and 3b were processed with the processing software.

### 2.3 FTIR Microspectroscopy: Composition Gradients

The three composition gradient films were fully mapped across the gradient direction (*x*-axis), and across 1.5 cm of the constant *y*-axis direction. Spectra were collected from every 400 μm across the gradient (the *x*-axis) and every 800 μm along the *y*-axis (film A: 152 × 21 matrix of spectra, total of 3192; film B: 164 × 21 matrix of spectra, total of 3444; film C: 154 × 17 matrix of spectra, total of 2618). The composition gradient maps were processed with the acquisition package in the same manner as the calibration maps in order to allow direct comparison of the data (see Figs. 5 to 7). The baselines were corrected between 1500 cm^−1^ and 1150 cm^−1^, the 1270 cm^−1^/1450 cm^−1^ peak area ratios were measured in the same spectral regions and the same color coding as the maps of the discrete composition films was used.

## 3. Results

In order to characterize the PLLA-PDLLA composition gradients with FTIR-RTM, we had to construct a calibration curve using spectra obtained from discrete blend films (0, 25, 50, 75, 100) % by mass fraction of PLLA/PDLLA. The spectra were analyzed by Method A, and the results were compared to these obtained from the high-throughput analysis (Method B). The average peak ratios obtained manually using Method A from four spectra (four yellow pixels) extracted from the homogenous map of the 50 % film (285 pixels out of 288 are yellow), agreed to within 0.3 % with the value concurrently obtained using the processing software (Method B) from the whole map (288 spectra). The average peak ratios obtained manually from five representative spectra (four different colors) extracted from the heterogeneous map of the 75 % PDLLA film was within 0.6 % of the value obtained with the processing software simultaneously from the map’s 120 spectra. The agreement in the average peak ratios obtained from the same maps (homogenous and heterogeneous) by Method A and Method B indicate that the processing package was able to correctly process data obtained by the acquisition package in a high-throughput fashion.

**Fig. 4:**
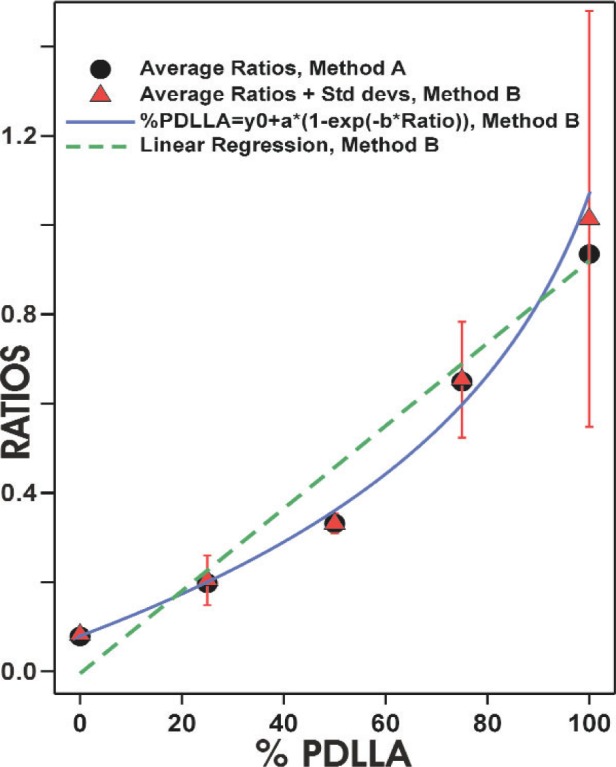
Overlaid averages of the peak ratios obtained by Method A, averages and standard deviations of the peak ratios obtained by Method B, the fitted linear regression line and the non-linear regression curve (Method B), versus % PDLLA (error bars represent the peak ratios uncertainty at each blend composition).

The averages of the peak ratios obtained by Method A and Method B were plotted against % PDLLA and lines were fit to the data, using linear regression. As can be seen in Fig. 4, the average ratios obtained by Method A and method B are nearly identical. The standard deviations (Method B) are especially large for 100 % PDLLA since these films were thinner (Table 1) with a lower SNR. The line that was fit to the data obtained by Method B is shown in Fig. 4. As can be seen, the line does not give an optimal fit to the data. This implies that the area of the 1270 cm^−1^ peak is not linearly dependent on blend composition. This is probably due to confounding effects from crystallinity on absorbance. It is well known that crystallinity can affect infrared absorbance [10]. Since PLLA is crystalline and PDLLA is amorphous, crystallinity will be varied in blends of these two polymers. It was shown with differential scanning calorimetry that the presence of PDLLA affected the crystallinity of PLLA when PLLA and PDLLA were blended [11]. With two factors affecting the PLLA-PDLLA blend spectra, composition and crystallinity; it is not surprising the area of the 1270 cm^−1^ peak is not linearly dependent on blend composition.

Non-linear regression was used to fit the data to a 3-parameter single exponential curve (Eq. 1).

$$\begin{array}{c} \%\text{PDLLA}=-19.9935+135.7455^{*}[1-\mathrm{Exp}(-2.0131^{*}\text{Ratio})]\\ R^{2}=0.99386799 \end{array}$$ (1)

As can be seen in Fig. 4, the curve fits the data quite well and will be used as a calibration curve for the determination of the compositions of the gradients.

**Fig. 5:**
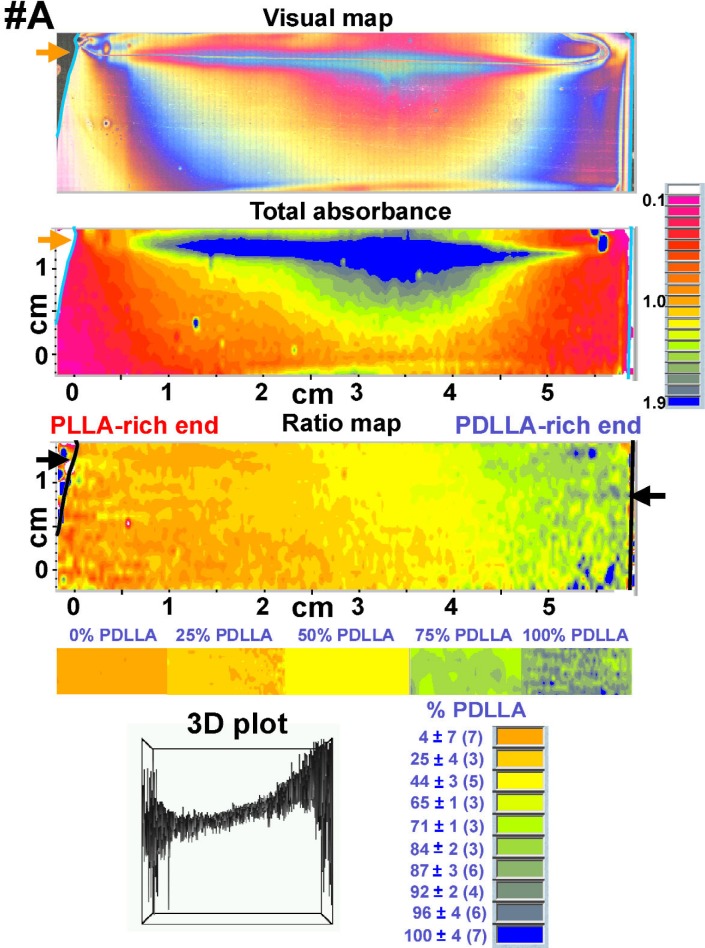
Visual map, total absorbance map (1500 cm^−1^ to 1155 cm^−1^ spectral region), ratio map (peak area at 1270 cm^−1^)/(peak area at 1450 cm^−1^) and 3D plot (arbitrary units) of PLLA-PDLLA composition gradient #A. The arrows on the visual and FTIR maps point to bare regions of the slide that are not covered with polymer film. The colors in these bare areas seen in the ratio maps are artifacts of ratioing spectra of the reflective glass substrate. The color bar, shown below the ratio map, was assembled from the middle parts of the ratio maps of the discrete composition films. The averages and standard deviations of the % PDLLA in the color code (bottom right panel) were calculated with the calibration curve. Numbers of spectra in each color group appear in parenthesis.

**Fig. 6:**
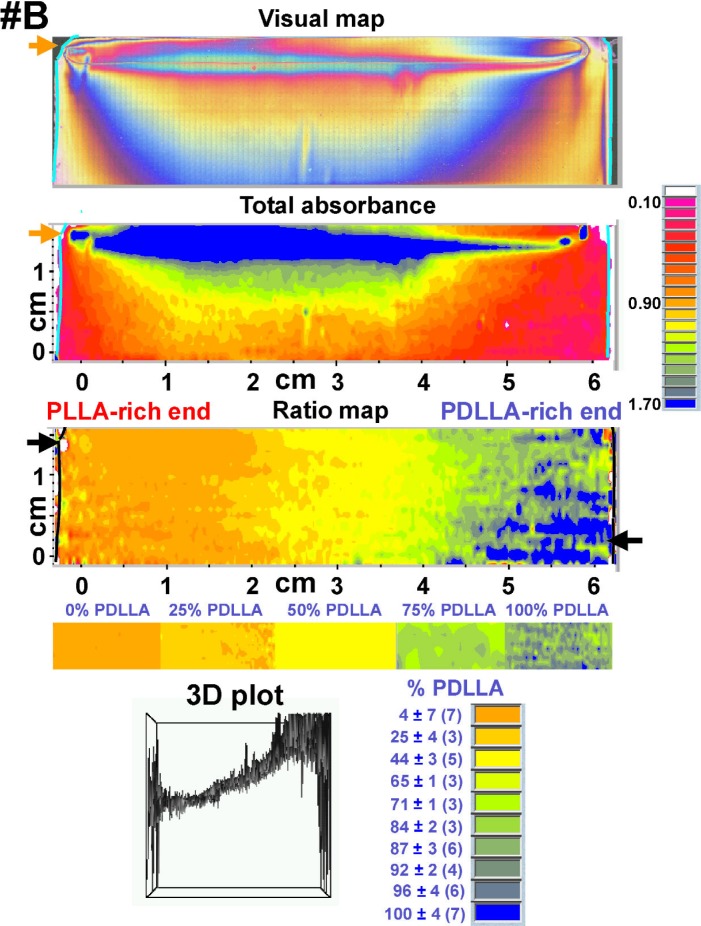
Visual map, total absorbance map (1500 cm^−1^ to 1155 cm^−1^ spectral region), ratio map (peak area at 1270 cm^−1^)/(peak area at 1450 cm^−1^) and 3D plot (arbitrary units) of PLLA-PDLLA composition gradient #B. The arrows on the visual and FTIR maps point to bare regions of the slide that are not covered with polymer film. The colors in these bare areas seen in the ratio maps are artifacts of ratioing spectra of the reflective glass substrate. The color bar, shown below the ratio map, was assembled from the middle parts of the ratio maps of the discrete composition films. The averages and standard deviations of the % PDLLA in the color code (bottom right panel) were calculated with the calibration curve. Numbers of spectra in each color group appear in parenthesis.

**Fig. 7:**
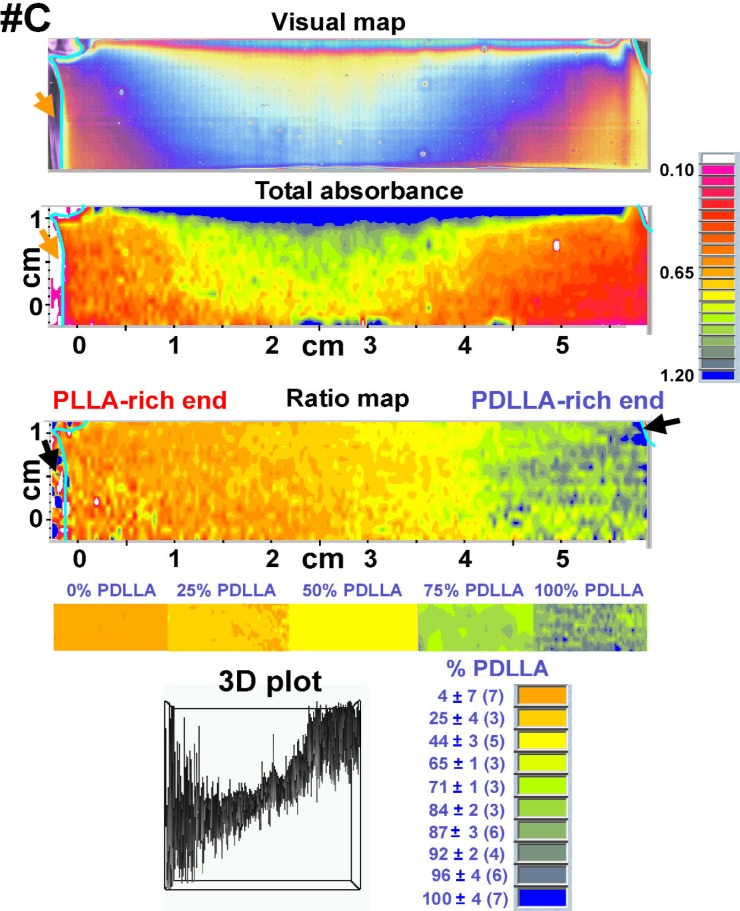
Visual map, total absorbance map (1500 cm^−1^ to 1155 cm^−1^ spectral region), ratio map (peak area at 1270 cm^−1^)/(peak area at 1450 cm^−1^) and 3D plot (arbitrary units) of PLLA-PDLLA composition gradient #C. The arrows on the visual and FTIR maps point to bare regions of the slide that are not covered with polymer film. The colors in these bare areas seen in the ratio maps are artifacts of ratioing spectra of the reflective glass substrate. The color bar, shown below the ratio map, was assembled from the middle parts of the ratio maps of the discrete composition films. The averages and standard deviations of the % PDLLA in the color code (bottom right panel) were calculated with the calibration curve. Numbers of spectra in each color group appear in parenthesis.

The calibration curve was used to calculate the composition of each pixel, from which the spectra for the manual measurements of the peak ratios were extracted, in order to assign the pixel color to % PDLLA. Two additional spectra were extracted from yellow-green and two from light green pixels in order to have an average and standard deviations for these colors also. The respective colors were regrouped, and compositions were assigned to each color (see color codes in Figs. 5–7). Currently, it is not possible to match the colors of the acquisition package maps with these of the processing software.

The results of the FTIR-RTM analyses of the three PLLA-PDLLA composition gradients {visual map, total absorbance map (1500 cm^−1^ to 1155 cm^−1^ spectral region), ratio map [(peak at 1270 cm^−1^)/(peak at 1450 cm^−1^)] and 3D plot, all obtained with the acquisition software} are shown in Figs. 5–7. The visual maps (top panels, Figs. 5–7) are composed of reflected light micrographs (622 μm × 466 μm each). The colors are not related to composition but are actual visual color created by differences in film thickness. Each visual map shows the part of the film that was mapped by the FTIR-RTM immediately after the video frames were captured. The total absorbance maps in the 1500 cm^−1^ to 1155 cm^−1^ region are shown in the middle panels of Figs. 5–7, while the 1270/1450 peak ratio maps are shown in the lower panels of Figs. 5–7. The 3D plots in the bottom of the figures show the peak ratios of each point in the map.

The entire length of each film in the gradient direction (*x*-axis) and most of each film in the direction perpendicular to the gradient (*y*-axis) were mapped in order to get as much information as possible. The upper parts of each of the three composition gradient films were included in the maps to allow direct comparison between the films and to have reference markers on each map for easy and accurate location when the same films will be used with other techniques for additional characterization. Comparison between the visual maps shown in Figs. 5–7 indicates the resemblance between the three gradient films. The matching absorbance (1500 cm^−1^ to 1155 cm^−1^) maps show that the total absorbance of the films is proportional to film thickness as can be seen when the visual map and the absorbance map are compared for each of the gradients (Figs. 5–7). The absorbance maps indicate that the films are thicker in the top middle part (blue represents the highest absorbance and pink represents the lowest) and become thinner towards the right and left edges and the bottom, most probably following the flow coating process direction. In order to normalize for the thickness, the area of the 1270 cm^−1^ peak, whose intensity increases with higher PDLLA concentration, was divided by the area of the relatively constant 1450 cm^−1^ peak. This normalization compensated for the differences in film thickness and clearly showed the gradients in the blend composition from left to right. In these figures orange indicates the lowest % PDLLA and blue represents the highest % PDLLA concentration. A definite trend from orange, in the PLLA-rich left edge of the gradient, to blue, in the PDLLA-rich right edge, is seen in all the three gradient films that were mapped (Figs. 5–7, ratio maps). The 3D plots in the bottom panels of Figs. 5–7 show the peak ratios of each point in the map across the films and indicate the achievement of continuous gradients in the three films. The higher presence of spikes in the left and right sides of the plots originated from erroneous peak ratios from spectra taken from the bare substrate outside the films, and partially from spectra taken from the thinner edges of the films, especially at the PDLLA side, resulting in higher noise from atmospheric water. The similarities among the visual, absorbance and peak ratio maps and the 3D images of the three films that were mapped show the qualitative reproducibility of the gradient composition preparation procedure, the flow coating technique and the FTIR-RTM mapping method.

## 4. Discussion

Previous studies have shown that FTIR microspectroscopy in reflectance mode (FTIR-RM) is capable of qualitatively and quantitatively characterizing the chemical properties of optically thick polymer specimens [12]. FTIR-RM mapping has been used to determine the distribution of the different chemical components in human gallstones [13], to characterize the minerals in pisoliths [14], the mineral and collagen in tooth dentin [15] and the mineral and resins in dental composites [16]. FTIR-RM mapping was utilized recently as a high-throughput method for determination of the temperature gradient curing of epoxy film (≈ 400 μm thick) [17]. Each of these studies mentioned above was performed on thick specimens and only the surface properties were determined. FTIR-RTM was used for the first time in this study to characterize the polymer composition gradients in thin films (≈ 150 nm to 400 nm).

Only a few reports describing the characterization of thin polymeric films with FTIR could be found in the literature. Transmission FTIR microscopy was used to determine the distribution of various transition-metal complexes embedded in thin pressed film fragments of polystyrene, poly(methyl methacrylate) and polystyrene-polyacrylonitrile copolymer, that were placed on KBr discs [18]. FTIR microscopy has also been used to verify homogeneity in polyimide thin films that were deposited on alkali halides substrates [19]. Combinatorial studies using FTIR were carried out mainly in the catalysis research field. FTIR imaging was used to monitor adsorbed CO monolayers prepared on various individual support catalyst pellets and to follow the reaction products in the gas phase. Multiple samples were measured simultaneously [20]. A high-throughput analysis in catalysis using transmission infrared spectroscopy was reported recently [21]. A newly designed holder made of silicon wells and wafer that can hold 48 different catalysts was developed for automated FTIR spectroscopic characterization of combinatorial copolymers composition. FTIR spectroscopy and a commercially available high-throughput FTIR system were used to determine enantiomeric purity [22]. This method is important for combinatorial asymmetric catalysis. Recently, a new technique, electrochemical *in situ* microscope FTIR reflection spectroscopy (MFTIRS) [23] was used to study the surface properties of combinatorial nanostructured Ru films electrochemically deposited on the individually addressable arrays of platinum microelectrodes. This technique enabled the authors to rapidly acquire an *in situ* FTIR spectral library of CO adsorbed on different nanostructured Ru films and at various electrode potentials.

Although there are no reports in the literature on characterizing PLLA or PDLLA with FTIR microspectroscopy, there are many studies using traditional FTIR spectroscopy. For example, a purified copolymer of PDLLA and polyethylene glycol (designed as drug delivery system) was dissolved in chloroform, coated on KBr tablets to form thin films and measured by an FTIR spectrophotometer [24]. Solutions of chloroform dissolved PDLLA homopolymers (5 % mass fraction per volume) with different molecular weights were spread on KBr tablets to form thin films, and their spectra were recorded with a FTIR spectrophotometer [25]. The miscibility of PDLLA and poly(p-vinylphenol) (PVPh) blends were studied by FTIR spectroscopy. Tetrahydrafuran solutions of the blends were cast on KBr disks, spectra were obtained with a FTIR spectrophotometer [26] and FTIR spectroscopy was used to measure the composition. The composition of a PDLLA-PCL (PCL, poly(epsilon-caprolactone)) composition gradient (0.3 μm to 1 μm thick) coated on a sapphire substrate was characterized by taking traditional spectra every few mm across a gradient [5].

The studies mentioned above are different from the system reported in this study in which composition gradients and discrete control blends (0, 25, 50, 75, 100) % by mass fraction were deposited directly on low-e reflective IR microscope substrates and characterized by FTIR-RTM. Further, the chemical composition along the gradients was determined by a calibration curve constructed from known discrete composition controls.

Three composition gradients of PLLA and PDLLA blends were created on reflective substrates and were characterized with FTIR-RTM. The formation of the composition gradients was confirmed qualitatively by the FTIR ratio maps and quantitatively by processing the acquisition software maps in the processing software and determining the position-resolved composition values with the calibration curve. The results show that the gradients were present and the methods used to characterize them were consistent and reproducible from sample to sample.

The technique reached its detection limits when extremely thin PDLLA-rich regions were measured. The absorptions of the atmospheric water interfered with the low intensity of the 1450 cm^−1^ peak and caused larger errors in the calculated peak ratios, resulting in artificial heterogeneity in the 100 % PDLLA and the 75 % PDLLA discrete film maps used for the calibration curve. The accuracy in the composition determination of the PDLLA-rich regions in the gradient films was also reduced because of the low intensity of the 1450 cm^−1^ peak in these thin regions. Efforts to prepare thicker gradient films are in progress.

Processing the FTIR maps with Method A allows for qualitative comparisons between various variables and maps, and manually processing individual representative spectra supplies a semi quantitative comparison. The use of the new high-throughput approach (Method B) however, improved the quantification of the results and saved enormous amounts of processing time. Additional efforts will be devoted to improving the integration of both software packages and to match the colors of the acquisition software maps to those acquired in the processing software.

The non-destructive, non-contact nature of the FTIR-RTM enables further determination of additional properties on the same chemically-characterized specimens. An additional advantage of the current system is the capability to obtain parallel visual and IR maps with the same microscope, allowing the user to evaluate the thickness of the films.

FTIR-RTM mapping was used here for the first time to map and determine the gradient composition of thin films of polymer blends flow coated on low emissive reflective glass slides. This resulted in reflection-transmission spectra enabling characterization of the full depth of the film and not just the surface, as was the case in several earlier studies with FTIR-RM [13–17]. The combination of the low-e substrate and the FTIR-RM provides the quality of transmission FTIR microscopy and the simplicity of reflectance FTIR. The low-e glass is easy to use, can be tailored to large specimens and is resistant to both organic solvents and water. The technique was adapted to the analysis of macro size samples (7 cm × 2 cm) of nanoscale film thicknesses (100 nm to 400 nm). The results of this study demonstrated the capability of the high-throughput technique FTIR-RTM to determine compositions of large combinatorial libraries of thin films of polymer blends.

## Figures and Tables

**Table 1:** Averages and standard deviations of the 1450 cm^−1^ peak areas obtained from the discrete films (*n* is the number of spectra averaged)

	% PDLLA	*n*	Avg 1450	Std 1450
Fig. 2a	0	1170	1.7	1.2
Fig. 2b	25	780	0.311	0.173
Fig. 2c	50	288	0.643	0.170
Fig. 2d	75	120	0.112	0.016
Fig. 2e	100	800	0.060	0.018
Fig. 3b	100	1802	0.057	0.025
